# Relationship between residual visual field and full-field stimulus testing in patients with late-stage retinal degenerative diseases

**DOI:** 10.1038/s41598-024-53474-6

**Published:** 2024-02-02

**Authors:** Daiki Sakai, Tadao Maeda, Midori Yamamoto, Satoshi Yokota, Akiko Maeda, Yasuhiko Hirami, Makoto Nakamura, Yasuo Kurimoto, Michiko Mandai

**Affiliations:** 1 Department of Ophthalmology, Kobe City Eye Hospital, 2-1-8 Minatojima Minamimachi, Chuo-ku, Kobe, Hyogo 650-0047 Japan; 2 Department of Ophthalmology, Kobe City Medical Center General Hospital, Kobe, Japan; 3 Department of Surgery, Division of Ophthalmology, Kobe University Graduate School of Medicine, Kobe, Japan

**Keywords:** Hereditary eye disease, Retinal diseases, Vision disorders

## Abstract

This study aimed to investigate how the extent and central/peripheral location of the residual visual field (VF) in patients with late-stage inherited retinal diseases (IRDs) are related to retinal sensitivity detected using full-field stimulus testing (FST). We reviewed the results of Goldmann perimetry and FST from the medical records of patients with IRDs whose VF represents central (within 10°) and/or peripheral islands, or undetectable. In total, 19 patients (19 eyes) were analyzed in this study. The median value of residual VF area was 1.38%. The median values of rod and cone sensitivities were  − 14.9 dB and 7.4 dB, respectively. Patients with only the peripheral island (− 33.9 dB) had better median rod sensitivity than other groups (only central,  − 18.9 dB; both,  − 3.6 dB). VF area significantly correlated with rod sensitivity (r = − 0.943, *p* = 0.005) in patients with only peripheral island, but not with cone sensitivity. Peripheral VF islands were significant contributors to FST results, especially rod sensitivity. With reduced or loss of central vision, the extent of residual peripheral VF significantly affected rod sensitivity, suggesting that FST can be useful in quantitatively estimating the overall remaining vision in patients with late-stage IRD.

## Introduction

The worldwide prevalence of inherited retinal diseases (IRDs) is approximately 1/4,000 individuals, making IRDs the most common cause of blindness in developed countries^1^; however, no definitive treatment for IRDs has been established. If patients are diagnosed with IRDs, progressive retinal degeneration is unavoidable, which leads to irreversible severe vision loss in the later stages of the disease. In recent years, emerging therapies, including stem cell-based^2^ and optogenetic therapies^3^, are expected to provide an opportunity to restore vision in patients with late-stage IRDs.

For developing therapy for late-stage IRDs, an important issue is the measurement of visual outcomes. Visual function evaluation in late-stage IRDs is challenging as severely impaired vision makes traditional examinations tough. Full-field stimulus testing (FST) is a specialized visual examination test for severe IRDs, which can quantitatively evaluate residual retinal sensitivity in the eyes with minimal visual function^4,5^. Previous studies have shown the correlations between FST results and accepted retinal anatomical or functional parameters, such as ellipsoid zone width on optical coherence tomography (OCT)^6^, hyperautofluorescent ring diameter on fundus autofluorescence (FAF)^6^, amplitude of cone electroretinogram^6,7^, or retinal sensitivities on static perimetry and microperimetry^7^, suggesting that FST can be an alternative method to evaluate visual outcomes in patients with late-stage IRDs. Several severe IRD clinical trials have already used FST as an outcome measure^8–11^. In theory, FST represents the retinal sensitivity of the best-functioning focal region irrespective of location. This principle of examination absolves patients from steady fixation, while location and extent of residual visual function are less considered. Patients with late-stage IRDs typically have a small area of central vision remaining after centripetal progression, but some patients have peripheral islands of vision^12,13^. We previously reported relatively good FST results in a patient with such peripheral island^14^. So far, there is no study assessing the relationship between the location of preserved retina and FST results. Does peripheral vision functionally substitute the central vision when tested by FST? Whether the extent of the residual visual field (VF) has a quantitative effect on FST results is another focus of interest. By answering these questions, our interpretation of FST results will become more profound, which helps us to use it practically. This study aimed to investigate the relationship between the central/peripheral location or extent of residual VF islands and retinal sensitivity detected using FST in patients with late-stage IRDs.

## Methods

### Study design

This study adhered to the principles of the Declaration of Helsinki and was approved by the Medical Ethics Committee of the Kobe City Medical Center General Hospital (Kobe, Japan). All patients provided written informed consent to participate in this study.

### Patients

We reviewed the medical records of 41 eyes from 23 patients with late-stage IRDs (best-corrected visual acuity [BCVA] of ≤ 0.1 in decimal units) who underwent Goldmann perimetry and FST with blue and red stimulation (within 7 months) at the Kobe City Eye Hospital between March 2019 and January 2023. Only patients with a VF representing isolated central (within 10°) or peripheral (outside of 10°) islands or both based on Goldmann perimetry were included in this study. Additionally, patients with no detectable VF on Goldmann perimetry were included. If the patients underwent several sessions of FST, the earlier results were used. If both eyes of one patient were eligible (16 patients), the better eye with respect to the extent of visual field was selected for statistical analyses to account for inter-eye correlation.

### Full-field stimulus testing (FST)

After a brief introduction (approximately 5 min), the FST was performed using an Espion system with software version V6.63.26 (Diagnosys LLC, MA, USA) in a completely dark room. The patients were dark-adapted for 45 min, and the pupils were dilated using mydriatic eye drops before the examination. Both eyes were independently examined by shielding the non-tested eyes. Blue (448 nm) and red (627 nm) light stimuli from the ColorDome™ stimulator were used to provide full-field light to the patients. The baseline intensity of 0 dB was set at 0.01 cd·s/m^2^. The available intensity ranged from   − 75 dB to + 30 dB (from dim to bright), and the starting intensity was 0 dB. The patients were provided a two-button box with yes/no buttons and were forced to answer whether they perceived light after a beep followed by a stimulus. The Weibull function was used to define the actual threshold while considering false positives and negatives^15,16^. A single trial per eye was performed, with 2 min of interval between trials. Quality assessment was manually performed by two ophthalmologists (D.S. and T.M.). Unreliable data was excluded.

### Interpretation of FST results

The difference between the thresholds for blue and red stimuli was used to determine photoreceptor subtype mediation based on previous knowledge^5^. The rod-mediated threshold is indicated when the difference is > 19.3 dB, whereas the cone-mediated threshold brings comparable thresholds (difference < 3.1 dB). When the difference was within these limits, the patient was considered to have a mixed-mediated threshold. The blue FST result was used as rod sensitivity if the patient has rod or mixed mediation. Similarly, the red FST result is available as cone sensitivity if the patient has a cone or mixed mediation. Additionally, retinal sensitivity was determined as the better one of the rod and cone sensitivities and was used for exploratory purposes. Supplementary Table S1 shows the profiles and results of each participant.

### Goldmann perimetry

Kinetic VF testing was performed using Goldmann perimetry (Haag Streit, Bern, Germany). The background luminance was set at 31.5 asb, and the V4e, I4e, I3e, I2e, and I1e targets were tested sequentially. Since most patients in this study could not see the I4e, test targets between V4e and I4e were measured appropriately. Eye gaze was carefully monitored during the VF testing, especially in patients with reduced central vision, and the examiner encouraged patient fixation as needed. The results were scanned and registered using the ImageJ software (National Institute of Health, MD, USA). Residual VF area was determined as follows: First, an area 60° superiorly, 90° temporally, 70° inferiorly, and 70° nasally, referring to normal VF extension, was trimmed from the original chart^17^. The accumulation of the V4e isopter area was then measured, and the percentage of the total trimmed area was defined as the VF area (%). This measurement was based on the agreement between the two examiners (D.S. and M.Y.), and the average value of the two measurements was used. The final isopter (for the most dim and small target) detected in the VF test was recorded for each participant.

### Visual acuity

BCVA was measured monocularly using the Landolt C charts under the normal room lighting condition (approximately 300 lx). Light perception was initially determined by the patient’s response to penlight stimulation under room lighting condition. If the response was absent, examiner repeated the same procedure under the dim-room condition (in the room for either ocular imaging, perimetry, or electrophysiology). Only patients with a BCVA of ≤ 0.1 (decimal) were included. We categorized the patients into the following four groups according to BCVA: > 0.01, 0.01/counting fingers, hand motion, and light perception. Additionally, BCVA was converted to logarithm of the minimum angle of resolution (logMAR) equivalent for quantitative assessment. Extremely low BCVA was converted to a value of 2.0 for counting fingers, 2.3 for hand motion^18^, and 2.7 for light perception^19^.

### OCT

Spectral-domain OCT images were obtained using Spectralis (Heidelberg Engineering, Heidelberg, Germany). Central foveal thickness (CFT) was measured as the distance from the vitreoretinal interface to the inner border of the retinal pigment epithelium (RPE) at the fovea. The average CFT values obtained from the horizontal and vertical OCT images were used in the analyses. The length was measured using the “caliper” function of the internal software (Heidelberg Eye Explorer version 6.12.3.0). Eyes with apparent vitreoretinal confounding diseases that affected foveal morphology (vitreomacular traction syndrome, n = 2; cystoid macular edema, n = 1) were excluded from this measurement.

### Statistical analyses

The normality of the data distribution was tested using the Kolmogorov–Smirnov test. Between-group analyses were performed using the Kruskal–Wallis test. Correlation analyses were performed using Spearman’s rank correlation coefficient. All statistical analyses were performed using the SPSS software package (version 28; SPSS Inc., Chicago, IL, USA). The statistical significance of all tests was set at *p* ≤ 0.05.

### Ethics approval

All procedures performed in studies involving human participants were in accordance with the ethical standards of the Kobe City Medical Center General Hospital (Kobe, Japan) and the principles of the 1964 Declaration of Helsinki and its later amendments or comparable ethical standards.

### Consent to participate

All patients provided written informed consent to participate in this observational study before undergoing the FST.

## Results

Thirty-five eyes of 19 patients were included in this study, and 19 eyes of 19 patients were analyzed by selecting the better eye with regard to the VF area. Among these participants, 17 patients were diagnosed with retinitis pigmentosa (RP), one patient had Stargardt disease, and the remaining one patient had Bietti crystalline dystrophy. Ten of the 17 patients with RP underwent genetic testing, and *RPGR* variants were identified in three patients; *RHO* and *MERTK* variants were each identified in one individual, and the remaining five patients were not genetically identified. The patient with Stargardt disease was identified as having *ABCA4* variants. The patient with Bietti crystalline dystrophy did not undergo genetic testing and was diagnosed based on clinical findings. Among 16 patients whose both eyes were included in this study, half of the patients (eight patients) had some asymmetrical VF findings including the differences in the location of VF island (four patients) or the differences of 1% or more in VF area (six patients).

The characteristics of 19 patients (19 eyes) are listed in Table 1. The mean age (standard deviation) at examination was 60.8 (12.3) years. Regarding the location of the VF island, eight patients had only peripheral islands, five patients had only central islands, and five patients had both peripheral and central islands. The VF was undetectable in one patient. The median value of VF area was 1.38% (range, 0–12.4%). The photoreceptor mediation of FST was determined as rod mediation in seven patients, cone mediation in eight patients, and mixed mediation in four patients. As a result, rod and cone sensitivities were obtained for 11 and 12 patients, respectively. The median value of rod sensitivity was  − 14.9 (range,  − 48.8 to 3.9) dB, and that of cone sensitivity was 7.4 (range, 0.6 to 14.6) dB. The retinal sensitivity was obtained as the better one of rod and cone sensitivities for all 19 patients, and the median value was 0.6 (range,  − 48.8 to 14.6) dB.

**Table 1:** Characteristics of study subjects.

	19 eyes from 19 patients
Age (years), mean (SD)	60.8 (12.3)
Sex (Female/Male), n	9/10
Lens status (Cataract/IOL), n	10/9
BCVA (> 0.01/0.01 or FC/HM/LP), n	4/4/8/3
GP	
Only peripheral island, n (%)	8 (42.1%)
Only central island, n (%)	5 (26.3%)
Both peripheral and central islands, n (%)	5 (26.3%)
Undetectable, n (%)	1 (5.3%)
Smallest target size (I, II, III, IV, none), n	1/1/12/4/1
VF area (%)	
Median (IQR)	1.38 (7.65)
Range	0–12.4
OCT	
CRT (μm), median (IQR)	74.5 (57.8)
FST	
Mediation (R/C/M), n	7/8/4
Rod sensitivity (dB)	
Median (IQR)	− 14.9 (37.4)
Range	− 48.8 to 3.9
Cone sensitivity (dB)	
Median (IQR)	7.4 (6.6)
Range	0.6 to 14.6
Retinal sensitivity (dB)	
Median (IQR)	0.6 (34.5)
Range	− 48.8 to 14.6

SD, standard deviation; IOL, intraocular lens; BCVA, best-corrected visual acuity; FC, finger count; HM, hand motion; LP, light perception; GP, Goldmann perimetry; VF, visual field; IQR, interquartile range; OCT, optical coherence tomography; CRT, central retinal thickness; FST, full-field stimulus testing; R, rod; C, cone; M, mixed.

### Relationship among visual acuity, isopter of Goldmann perimetry, OCT measurements, and FST

**Figure 1:**
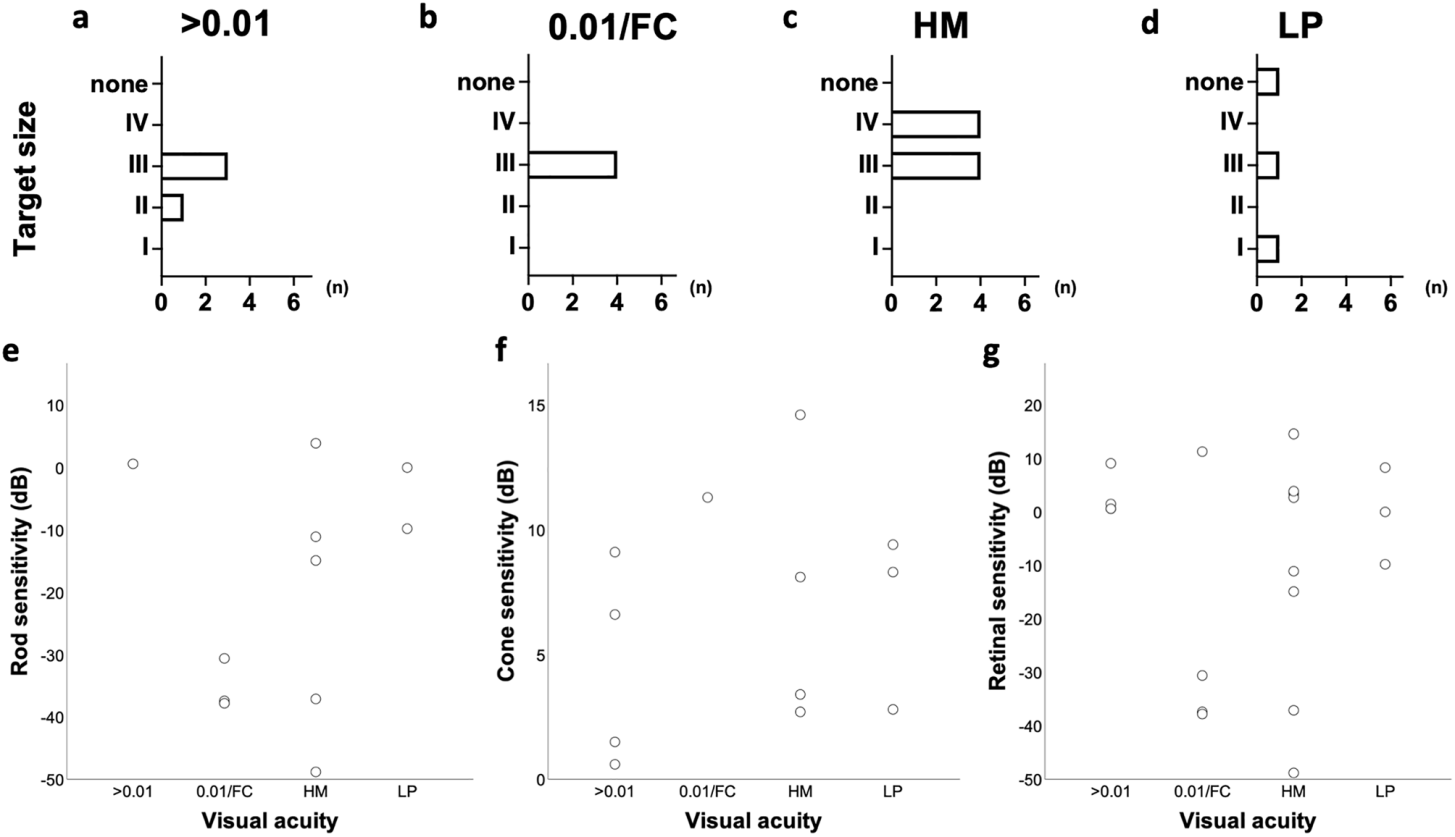
(**a**–**d**) Distribution of the smallest target size on the Goldmann perimetry of I to IV in eyes with best-corrected visual acuity (BCVA) of  > 0.01 (**a**), 0.01/finger count (FC) (**b**), hand motion (HM) (**c**), and light perception (**d**). (**e**–**f**) Distribution of rod (**e**), cone (**f**), and retinal (**g**) sensitivities detected by full-field stimulus testing in four BCVA groups.

**Figure 2:**
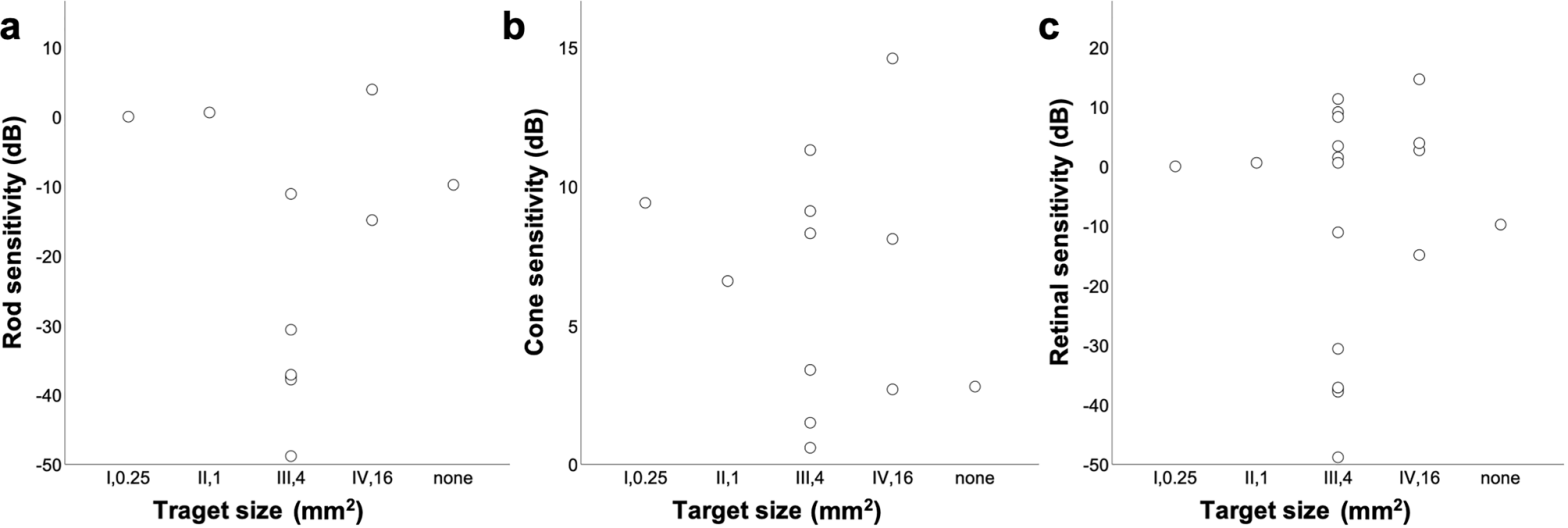
Distribution of rod (**a**), cone (**b**), and retinal (**c**) sensitivities detected using full-field stimulus testing in eyes with the smallest target size on the Goldmann perimetry of I to IV.

The patients were first divided into four groups according to their BCVA: > 0.01 (four patients), 0.01/counting finger (four patients), hand motion (eight patients), and light perception (three patients). In Goldmann perimetry, most patients exhibited the isopter only for maximum luminance level (4e), except for two patients who had the isopter for 4c level, whereas the detectable isopters for different target size ranged from I to IV size. Figure 1 summarizes the distribution of the target size of final isopter detected using the Goldmann perimetry and FST results in the four BCVA groups. There was no significant difference in the distribution of the FST results between BCVA groups. LogMAR BCVA was correlated with rod (r = 0.246) and cone (r = 0.356) sensitivities, but did not reach statistical significance (*p* = 0.465 and 0.257, respectively). No correlation was noted between logMAR BCVA and retinal sensitivity (r = − 0.003, *p* = 0.990). Figure 2 shows the relationship between distribution of FST results and the target size of final isopter in Goldmann perimetry. The distribution of FST results did not differ significantly between the groups. Additionally, there was no significant correlation between the target size (converted to mm^2^) and FST results (r =  − 0.048, *p* = 0.895 for rod, r =  − 0.010, *p* = 0.977 for cone, and r = 0.266, *p* = 0.286 for retinal sensitivity, respectively). The median value of CFT obtained from OCT images was 74.5 (range, 14.5–185.5) μm. There was no significant correlation between the CFT and FST results (Supplementary Fig. S1).

### Location of visual field island and photoreceptor subtype mediation

Table 2 shows the distribution of the location of the VF island and the photoreceptor subtype mediation of the FST. Five out of the eight (62.5%) patients with only peripheral islands had rod-mediated thresholds, and three out of five (60.0%) patients with only central islands had cone-mediated thresholds.

**Table 2:** Distribution of patients by location of visual field island and photoreceptor mediation of full-field stimulus testing.

		Photoreceptor mediation of FST		
		Rod	Cone	Mixed
Location of visual field island	Only peripheral	5	2	1
	Only central	1	3	1
	Both	1	3	1
	None	0	0	1

FST, full-field stimulus testing.

### Relationship between residual visual field and retinal sensitivity on FST

**Figure 3:**
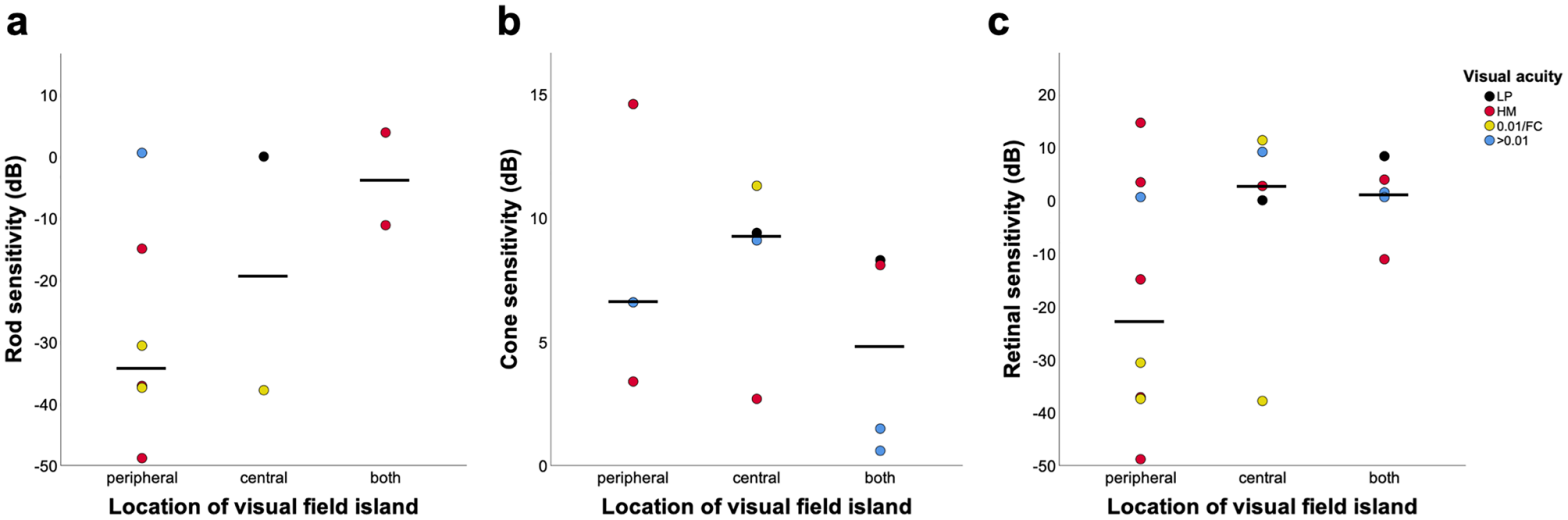
Dot plots showing the median and distribution of rod (**a**), cone (**b**), and retinal (**c**) sensitivities detected using full-field stimulus testing in eyes with only peripheral, only central, and both islands.

**Figure 4:**
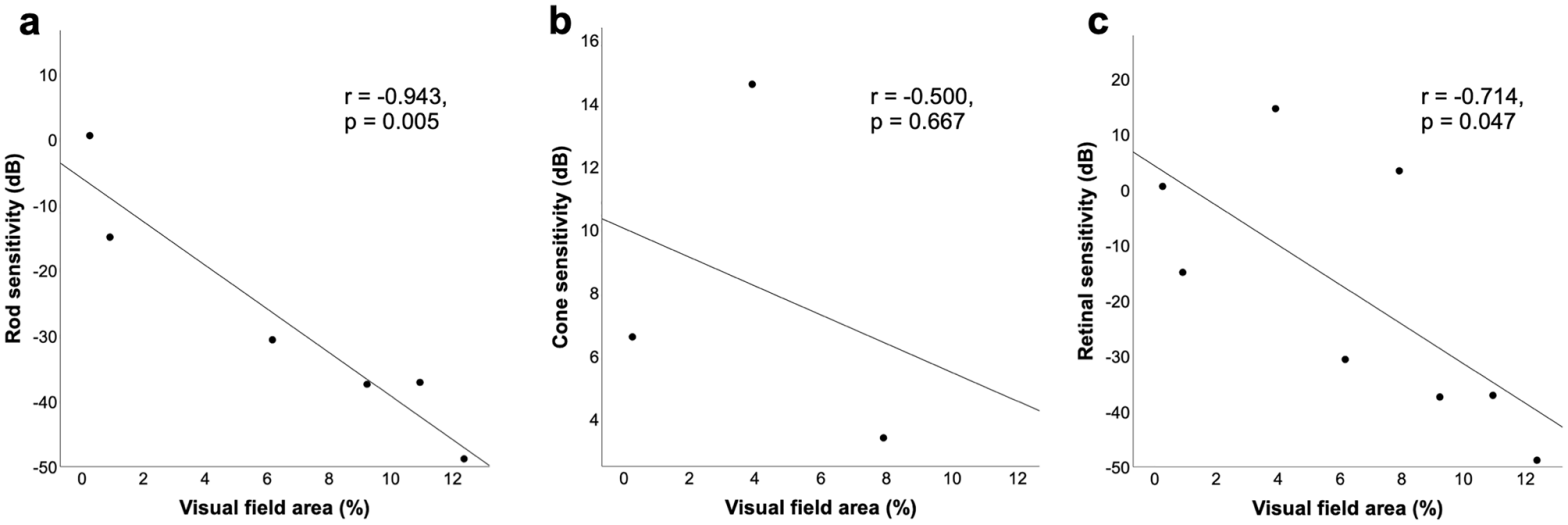
Scatterplots showing the relationship between visual field (VF) area and retinal sensitivity detected using full-field stimulus testing in patients with only peripheral VF island. (**a**) Relationship between the VF area and rod sensitivity. (**b**) Relationship between the VF area and cone sensitivity. (**c**) Relationship between the VF area and retinal sensitivity.

Figure 3 shows the distribution of FST results among the eyes in three groups according to the location of the VF island (the group with no detectable VF was eliminated, considering sample size, n = 1). The median rod sensitivities were  − 33.9 dB,  − 18.9 dB, and  − 3.6 dB in patients with only peripheral island, only central island, and both peripheral and central islands, respectively. The median retinal sensitivities were  − 22.8 dB, 2.7 dB, and 1.5 dB among the group of peripheral, central, and both, respectively. The median rod and retinal sensitivities were better in patients with only peripheral islands compared with those in the other groups, although there were no significant inter-group differences. Retinal sensitivity in patients with only peripheral island was more diverse compared with those in the other groups. There were no significant differences in the distribution of cone sensitivity between the groups. In the patient with no detectable VF, rod and cone sensitivity was  − 9.8 dB and 2.8 dB, respectively. As peripheral vision generally occupies a larger area compared with the central vision in advanced IRDs, we analyzed if the VF area had an effect on FST sensitivity for patients in peripheral group. The total VF area was significantly correlated with rod sensitivity (r = − 0.943, *p* = 0.005) and retinal sensitivity (r = − 0.714, *p* = 0.047), but not with cone sensitivity (r = − 0.500, *p* = 0.667) (Fig. 4). Additionally, among the five patients who had available data from both eyes with only peripheral island, the eye with larger VF area had better retinal sensitivity in four patients (Supplementary Table S2).

### Relationship between lens status and retinal sensitivity on FST

There were ten eyes with cataract, whereas remaining nine eyes were implanted with intraocular lens (IOL). Mean (standard deviation) age of patients with cataract (56.1 [5.3] years) was younger than that of patients with IOL (66.0 [15.7] years), although this difference was not statistically significant.

Median (interquartile range) VF area of patients with cataract (5.24 [9.00]%) was better than that of patients with IOL (1.06 [2.74]%), although this difference was not statistically significant. Median rod of patients with cataract (− 26.0 [33.9] dB) was better than that of patients with IOL (− 11.1 [34.5] dB), although this difference was not statistically significant. Median cone and retinal sensitivity were almost similar between patients with cataract and IOL (8.2 [6.4] and 4.7 [9.4] dB for cone sensitivity and − 3.2 [45.7] and 0.6 [23.0] dB, respectively). The IOL models were available from medical records of three patients, and all patients implanted blue light-filtering IOL (NX-70S: n = 1 [Santen, Osaka, Japan], CNW: n = 1 [Alcon Laboratories, Fort Worth, TX, USA], DCB00V: n = 1 [AMO, Santa Ana, CA, USA]).

### Representative cases

Goldmann perimetry and FST results of a representative case (E19, the right eye of a 71-year-old woman diagnosed with RP) are shown in Supplementary Fig. S2. Although she sometimes responded to V/4e stimuli, no isopter island was determined by Goldmann perimetry. The remaining retinal sensitivity was detected using FST (rod sensitivity,  − 14.2 dB; cone sensitivity,  + 2.0 dB). Small islands may be detectable somewhere, including the periphery, by careful follow-up VF testing. In contrast, another case (E3, the right eye of an 83-year-old man diagnosed with RP) warns a careful interpretation of the FST results (Supplementary Fig. S3). The patient had a lower peripheral VF island, with a calculated VF area of 5.60%. His FST results for blue and red stimuli were  + 3.0 dB and  − 6.6 dB, respectively. Because the threshold for red stimuli was better than that for blue, his results were determined as cone-mediated with a sensitivity of  − 6.6 dB. However, considering the expected advantage of peripheral vision preservation for rod sensitivity, careful evaluation is recommended at the next visit.

## Discussion

FST is a strong tool to detect the remaining retinal sensitivity in late-stage IRDs even in patients with no light perception during routine visual acuity test^14^. Late-stage IRDs sometimes show peripheral visual island other than typical central island, but how the different pattern of residual VF affects the FST results is unknown. In this study, we evaluated the effects of the central/peripheral location and extent of residual VF on retinal sensitivity detected by FST in late-stage IRDs.

Although the ability to distinguish rod and cone sensitivities by chromatic stimulus is a great advantage of the FST, it may not reflect the deteriorated physiological properties of photoreceptors in certain populations with late-stage IRDs. Thus, in the current study, in addition to the reported rod/cone sensitivity^5^, we tried integrating the FST results by defining “retinal sensitivity” as the better one of rod and cone sensitivities detected by FST, which reflect the best sensitivity of residual photoreceptor regardless of its type. We found a trend in the distribution of rod/cone mediation according to the location of the VF island. Rod mediation was dominant among patients with only peripheral islands, whereas cone mediation was dominant among patients with only central islands. Therefore, cone sensitivity of > 50% of the patients with only peripheral islands could not be obtained (5/8), and rod sensitivity was unavailable in the majority of the patients with central islands only (5/3). Available information from FST results may be different depending on the pattern of residual VF, reflecting heterogeneous pathology of IRDs.

Patients with late-stage IRDs have phenotypic heterogeneity according to different causal genes, and certain populations have a remaining peripheral vision^12,13^. Peripheral vision is crucial for spatial navigation^20^ and reading with appropriate training^21^, which justifies peripheral vision as a potential target for vision rescue or enhancement therapies. Routine assessment of IRDs should include the evaluation of peripheral vision. Goldmann perimetry is a feasible choice for detecting peripheral islands, but especially in patients without stable fixation, it is generally considered difficult to use for quantitative evaluation. Herein, our results showed a reasonable correlation between the rod/retinal sensitivity by FST and the VF area by Goldmann perimetry in patients with only peripheral VF island. This suggests that both the Goldmann perimetry and FST can be useful tools to quantitatively assess peripheral retinal vision. Specifically for rod or retinal sensitivity, peripheral VF preservation seems to be advantageous. In addition, the extent of the peripheral VF could affect retinal or rod sensitivity in patients with late-stage IRDs. In principle, the FST result shows a representative focal function of the most sensitive region of the retina but not an aggregate of contributions from all residual areas^4,5^. However, in our study, the final isopter in the Goldmann perimetry did not relate with FST results, and this is possibly due to the fact that the isopter groups for comparison here were based only on the size of stimuli (luminance was not diverse). Unlike rod or retinal sensitivity, cone sensitivity did not correlate with the extent of the VF. This might be partly due to the narrower range of cone sensitivities. The FST results for cone sensitivity could be biased as only patients with late-stage IRDs and severe central vision impairment were included.

Referring to other complementary examinations is useful for the interpretation of FST results. Recently, Ngo et al.^6^ showed that in patients with advanced RP, a better FST result was correlated with a better status of findings in posterior pole imaging, such as ellipsoid zone width on OCT and hyperautofluorescent ring diameter on FAF. Similarly, Dimopoulos et al.^22^ reported that FST results in patients with choroideremia were proportional to the extent of the preserved RPE area measured using posterior pole FAF images. These findings suggest that a broader central VF is advantageous for a higher sensitivity of the FST, which is reasonable considering typical centripetal progression of RP and choroideremia. Simultaneously, as shown in this study, the remaining peripheral vision in patients with late-stage IRDs significantly contributes to FST, especially in rod sensitivity. We propose that assessment of peripheral vision is an important complementary examination to the FST.

IOL was commonly designed to filter blue light, which could potentially affect the results of rod sensitivity obtained from blue light stimuli of FST. There was a trend of better rod sensitivity in patients with phakic eyes compared with those with IOL implanted eyes. It was possible that better rod sensitivity in patients with phakic eyes was due to severity of retinal diseases, supported by the younger age compared with patients with IOL, but the potential impact of blue-light filtering IOL on rod sensitivity on FST should be considered in further investigations.

In this study, we analyzed the FST results obtained from a single examination, whereas other studies usually used data from multiple repeated examinations. We currently use single FST in clinical practice owing to its good repeatability, as previously reported (within-session standard deviation < 2 dB)^5^. The patient with *MERTK*-RP (E35) had data available of the thrice repeated FST, and showed within-session differences < 2 dB, which support good repeatability of FST in late-stage IRDs. However, technical errors are a risk, such as in the abovementioned case (E3). Other limitations include the limited sample size and the retrospective nature of this study. Confirmation of our preliminary with a larger number of patients is warranted. Additionally, information regarding the causative gene was limited, although half of the participants underwent genetic testing. To understand the relationship between the underlying pathology and FST results, and how to interpret the rod and cone sensitivities obtained from chromatic stimulus in late-stage IRDs, a gene-by-gene assessment can be an intriguing research task.

## Conclusion

Peripheral VF islands are significant contributors to FST results, and the extent of the residual peripheral VF may affect the rod and retinal sensitivities of FST in patients with late-stage IRDs. Conversely, FST can be useful to quantitatively estimate the overall residual peripheral vision in the eyes with late-stage IRDs with reduced or loss of central vision.

### Supplementary Information

Supplementary Information.

## Data Availability

All data generated or analyzed during this study are included in this published article and its supplementary information file.
