# Supplementary material for: Relationship between residual visual field and full-field stimulus testing in patients with late-stage retinal degenerative diseases
Source: Sci Rep. 2024 Feb 2;14:2793. doi: 10.1038/s41598-024-53474-6 (PMC10837419; doi:10.1038/s41598-024-53474-6)

Relationship between residual visual field and full-field stimulus testing in patients with late-stage retinal degenerative diseases

Daiki Sakai[1,2,3*] MD, Tadao Maeda[1] MD, PhD, Midori Yamamoto[1] BA, Satoshi Yokota[1,2] MD, PhD, Akiko Maeda[1,2] MD, PhD, Yasuhiko Hirami[1,2] MD, PhD, Makoto Nakamura[3] MD, PhD, Yasuo Kurimoto[1,2] MD, PhD, Michiko Mandai[1,2] MD, PhD

[1]Department of Ophthalmology, Kobe City Eye Hospital, Kobe, Japan

[2]Department of Ophthalmology, Kobe City Medical Center General Hospital, Kobe, Japan

[3]Department of Surgery, Division of Ophthalmology, Kobe University Graduate School of Medicine, Kobe, Japan

Supplementary Information

Supplementary Table S1. Profiles and findings of included 35 eyes from 19 patients.

Eye ID	Analyzed eye	Patient ID	Age	Sex	Diagnosis	Causative gene	Eye	Lens status	BCVA	Blue	Red	Blue-Red	Mediation	Rod sensitivity	Cone sensitivity	Retinal sensitivity	VF area (%)	Final isopter	Peripheral island	Central island	Only peripheral island	Only central island	Both	None	Mean CRT	Confounding diseases
E1	No	P1	66	M	RP	RPGR	L	cataract	HM	11.3	11.5	0.2	C	NA	11.5	11.5	0.31%	Ⅳ/4e	No	Yes	No	Yes	No	No	156.5	
E2	Yes	P1	66	M	RP	RPGR	R	cataract	0.01	11.7	11.3	-0.4	C	NA	11.3	11.3	0.80%	Ⅲ/4e	No	Yes	No	Yes	No	No	NA	Cystoid macular edema
E3	No	P2	83	M	RP	NA	R	IOL	HM	3.0	-6.6	-9.6	C	NA	-6.6	-6.6	5.60%	Ⅲ/4e	Yes	No	Yes	No	No	No	84	
E4	Yes	P2	83	M	RP	NA	L	IOL	FC	-30.6	-5.5	25.1	R	-30.6	NA	-30.6	6.17%	Ⅲ/4e	Yes	No	Yes	No	No	No	185.5	
E5	No	P3	54	F	RP	NA	L	IOL	HM	-5.1	2.1	7.2	M	-5.1	2.1	-5.1	5.44%	Ⅳ/4e	Yes	Yes	No	No	Yes	No	89	
E6	Yes	P3	54	F	RP	NA	R	cataract	HM	1.6	3.4	1.8	C	NA	3.4	3.4	7.91%	Ⅲ/4e	Yes	No	Yes	No	No	No	108.5	
E7	No	P4	59	M	RP	RPGR	R	cataract	LP	7.2	7.6	0.4	C	NA	7.6	7.6	0.13%	Ⅲ/4e	No	Yes	No	Yes	No	No	27	
E8	Yes	P4	59	M	RP	RPGR	L	cataract	0.02	8.6	9.1	0.5	C	NA	9.1	9.1	0.25%	Ⅲ/4e	No	Yes	No	Yes	No	No	72.5	
E9	Yes	P5	47	M	RP	NA	L	cataract	LP	-9.8	2.8	12.6	M	-9.8	2.8	-9.8	0.00%	NA	No	No	No	No	No	Yes	65	
E10	No	P5	47	M	RP	NA	R	IOL	LP	4.8	13.4	8.6	M	4.8	13.4	4.8	0.00%	NA	No	No	No	No	No	Yes	100.5	
E11	No	P6	79	M	RP	NA	L	IOL	LP	5.5	8.4	2.9	C	NA	8.4	8.4	0.07%	Ⅳ/4e	Yes	No	Yes	No	No	No	133.5	
E12	Yes	P6	79	M	RP	NA	R	IOL	0.03	0.6	6.6	6.0	M	0.6	6.6	0.6	0.26%	Ⅱ/4e	Yes	No	Yes	No	No	No	117	
E13	No	P7	71	F	RP	NA	R	IOL	HM	8.0	6.7	1.3	C	NA	6.7	6.7	0.19%	Ⅳ/4e	No	Yes	No	Yes	No	No	70	
E14	Yes	P7	71	F	RP	NA	L	IOL	HM	6.2	2.7	-3.5	C	NA	2.7	2.7	0.22%	Ⅳ/4e	No	Yes	No	Yes	No	No	74.5	
E15	No	P8	50	M	RP	RPGR	L	cataract	LP	-13.0	n/a	NA(over 19.3)	R	-13.0	NA	-13.0	0.00%	NA	No	No	No	No	No	Yes	NA	Vitreomacular traction
E16	Yes	P8	50	M	RP	RPGR	R	cataract	HM	-14.9	7.4	22.3	R	-14.9	NA	-14.9	0.91%	Ⅳ/4e	Yes	No	Yes	No	No	No	NA	Vitreomacular traction
E17	No	P9	58	M	RP	unsolved	R	cataract	0.02	-35.4	-12.1	23.3	R	-35.4	NA	-35.4	2.92%	Ⅳ/4e	Yes	No	Yes	No	No	No	63.5	
E18	Yes	P9	58	M	RP	unsolved	L	cataract	0.01	-37.4	-13.9	23.5	R	-37.4	NA	-37.4	9.23%	Ⅲ/4e	Yes	No	Yes	No	No	No	76.5	
E19	No	P10	71	F	RP	NA	R	IOL	LP	-14.6	2.0	16.6	M	-14.6	2.0	-14.6	0.00%	NA	No	No	No	No	No	Yes	86	
E20	Yes	P10	71	F	RP	NA	L	IOL	0.01	-37.8	-13.0	24.8	R	-37.8	NA	-37.8	0.22%	Ⅲ/4c	No	Yes	No	Yes	No	No	95	
E21	No	P11	61	F	RP	RHO	R	IOL	LP	-0.5	4.4	4.9	M	-0.5	4.4	-0.5	0.15%	Ⅰ/4e	No	Yes	No	Yes	No	No	75.5	
E22	Yes	P11	61	F	RP	RHO	L	IOL	LP	0.0	9.4	9.4	M	0.0	9.4	0.0	0.28%	Ⅰ/4e	No	Yes	No	Yes	No	No	55.5	
E23	No	P12	59	M	RP	NA	R	cataract	HM	-46.5	-18.1	28.4	R	-46.5	NA	-46.5	9.02%	Ⅲ/4c	Yes	No	Yes	No	No	No	19.5	
E24	Yes	P12	59	M	RP	NA	L	cataract	HM	-48.8	-17.2	31.6	R	-48.8	NA	-48.8	12.37%	Ⅲ/4c	Yes	No	Yes	No	No	No	35.5	
E25	No	P13	54	F	RP	unsolved	R	cataract	0.01	2.4	9.2	6.8	M	2.4	9.2	2.4	6.12%	Ⅳ/4e	Yes	Yes	No	No	Yes	No	43	
E26	Yes	P13	54	F	RP	unsolved	L	cataract	HM	3.9	8.1	4.2	M	3.9	8.1	3.9	8.09%	Ⅳ/4e	Yes	Yes	No	No	Yes	No	14.5	
E27	No	P14	55	M	RP	unsolved	R	cataract	LP	11.3	11.4	0.1	C	NA	11.4	11.4	1.34%	Ⅴ/4e	Yes	No	Yes	No	No	No	44.5	
E28	Yes	P14	55	M	RP	unsolved	L	cataract	LP	4.9	8.3	3.4	C	NA	8.3	8.3	2.57%	Ⅲ/4e	Yes	Yes	No	No	Yes	No	54	
E29	No	P15	54	F	Stargardt disease	ABCA4	L	cataract	HM	-37.8	-13.0	24.8	R	-37.8	NA	-37.8	9.40%	Ⅳ/4e	Yes	No	Yes	No	No	No	78	
E30	Yes	P15	54	F	Stargardt disease	ABCA4	R	cataract	HM	-37.1	-12.8	24.3	R	-37.1	NA	-37.1	10.94%	Ⅲ/4e	Yes	No	Yes	No	No	No	75.5	
E31	No	P16	61	F	RP	unsolved	R	IOL	HM	-12.1	6.4	18.5	M	-12.1	6.4	-12.1	1.04%	Ⅳ/4e	Yes	Yes	No	No	Yes	No	127.5	
E32	Yes	P16	61	F	RP	unsolved	L	IOL	HM	-11.1	9.0	20.1	R	-11.1	NA	-11.1	1.06%	Ⅲ/4e	Yes	Yes	No	No	Yes	No	116.5	
E33	Yes	P17	58	F	RP	unsolved	R	IOL	HM	10.7	14.6	-3.9	C	NA	14.6	14.6	3.91%	Ⅳ/4e	Yes	No	Yes	No	No	No	139	
E34	Yes	P18	76	F	Bietti crystalline dystrophy	NA	R	IOL	0.03	0.6	1.5	0.9	C	NA	1.5	1.5	2.06%	Ⅲ/4e	Yes	Yes	No	No	Yes	No	72.5	
E35	Yes	P19	30	M	RP	MERTK	L	IOL	0.04	0.0	0.6	0.6	C	NA	0.6	0.6	1.38%	Ⅲ/4e	Yes	Yes	No	No	Yes	No	52.5	

BCVA, best-corrected visual acuity; VF, visual field; CRT, central retinal thickness; RP, retinitis pigmentosa; IOL, intraocular lens; FC, finger count; HM, hand motion; LP, light perception; R, rod; C, cone; M, mixed; NA, not available.

Supplementary Figure S1. Scatterplots showing the relationship between central retinal thickness (CRT) on optical coherence tomography and retinal sensitivity detected using full-field stimulus testing. (a) Relationship between the CRT and rod sensitivity. (b) Relationship between the CRT and cone sensitivity. (c) Relationship between the CRT and retinal sensitivity.

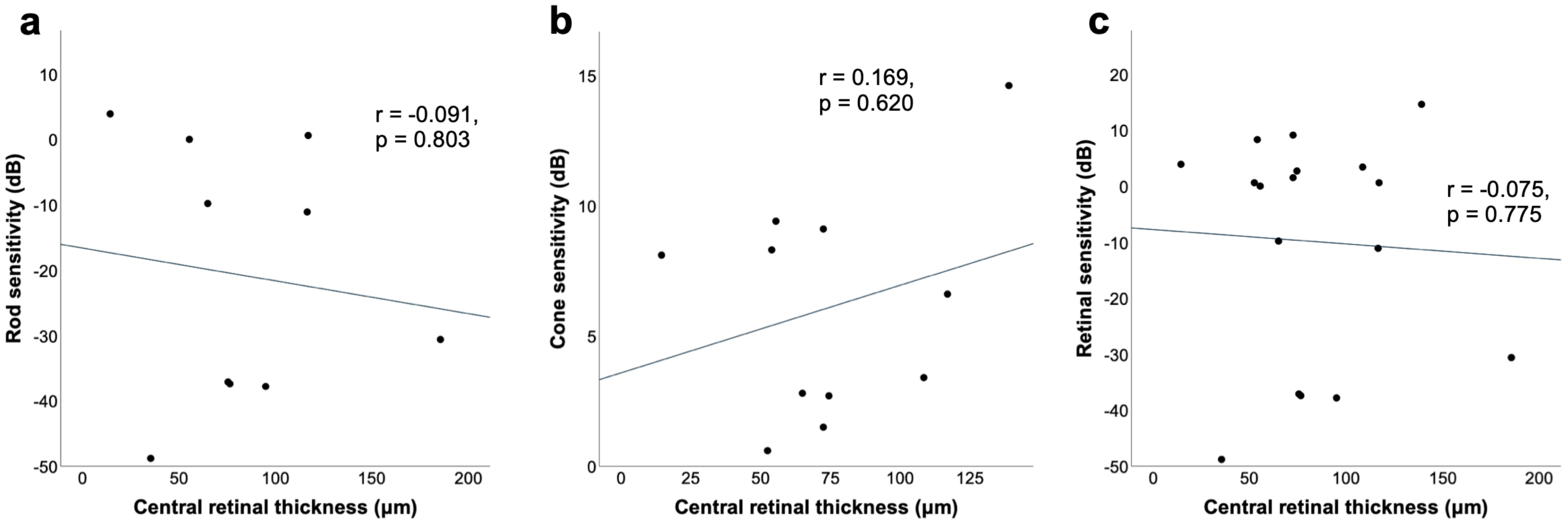

Supplementary Table S2. Visual field area and retinal sensitivity on full-field stimulus testing in patients whose both eyes had only peripheral visual islands.

Patient ID	Eye ID	Analyzed eye	Age	Sex	Diagnosis	Eye	VF area (%)		Retinal sensitivity (dB)		Consistency
P2	E3	No	83	M	RP	R	5.60%	worse	-6.6	worse	consistent
	E4	Yes	83	M	RP	L	6.17%	better	-30.6	better	
P6	E11	No	79	M	RP	L	0.07%	worse	8.4	worse	consistent
	E12	Yes	79	M	RP	R	0.26%	better	0.6	better	
P9	E17	No	58	M	RP	R	2.92%	worse	-35.4	worse	consistent
	E18	Yes	58	M	RP	L	9.23%	better	-37.4	better	
P12	E23	No	59	M	RP	R	9.02%	worse	-46.5	worse	consistent
	E24	Yes	59	M	RP	L	12.37%	better	-48.8	better	
P15	E29	No	54	F	Stargardt disease	L	9.40%	worse	-37.8	better	inconsistent
	E30	Yes	54	F	Stargardt disease	R	10.94%	better	-37.1	worse	

VF, visual field; RP, retinitis pigmentosa.

Supplementary Figure S2. Goldmann perimetry and full-field stimulus testing (FST) results of the right eye of a 71-year-old woman diagnosed with retinitis pigmentosa (E19).

E19

FST
Blue: -14.6 dB
Red: 2.0 dB

Mediation: Mixed
Rod: -14.6 dB
Cone: 2.0 dB

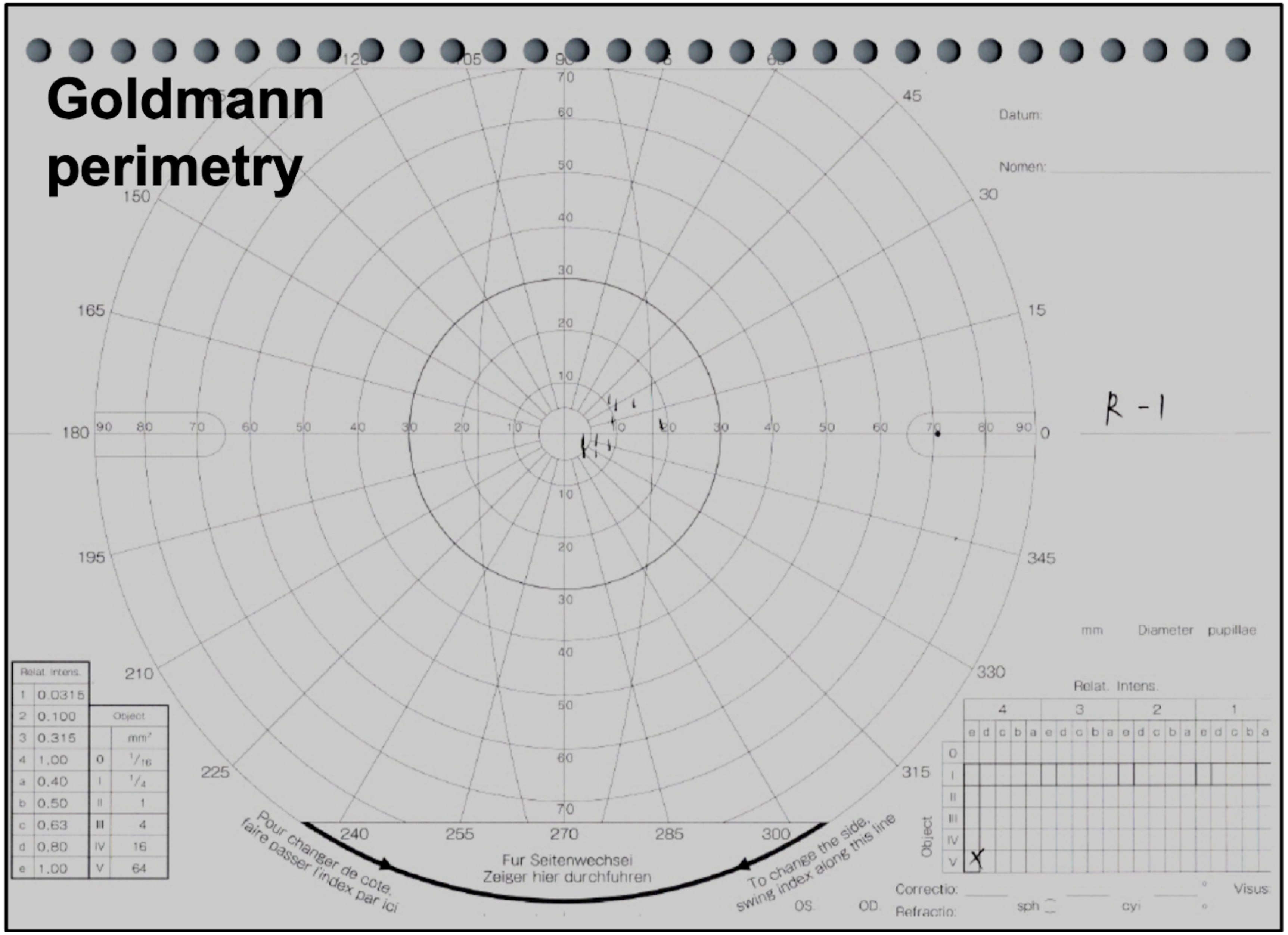

Supplementary Figure S3. Goldmann perimetry and full-field stimulus testing (FST) results of the right eye of a 83-year-old man diagnosed with retinitis pigmentosa (E3).

E3

FST
Blue: 3.0 dB
Red: -6.6 dB

Mediation: Cone
Rod: NA
Cone: -6.6 dB

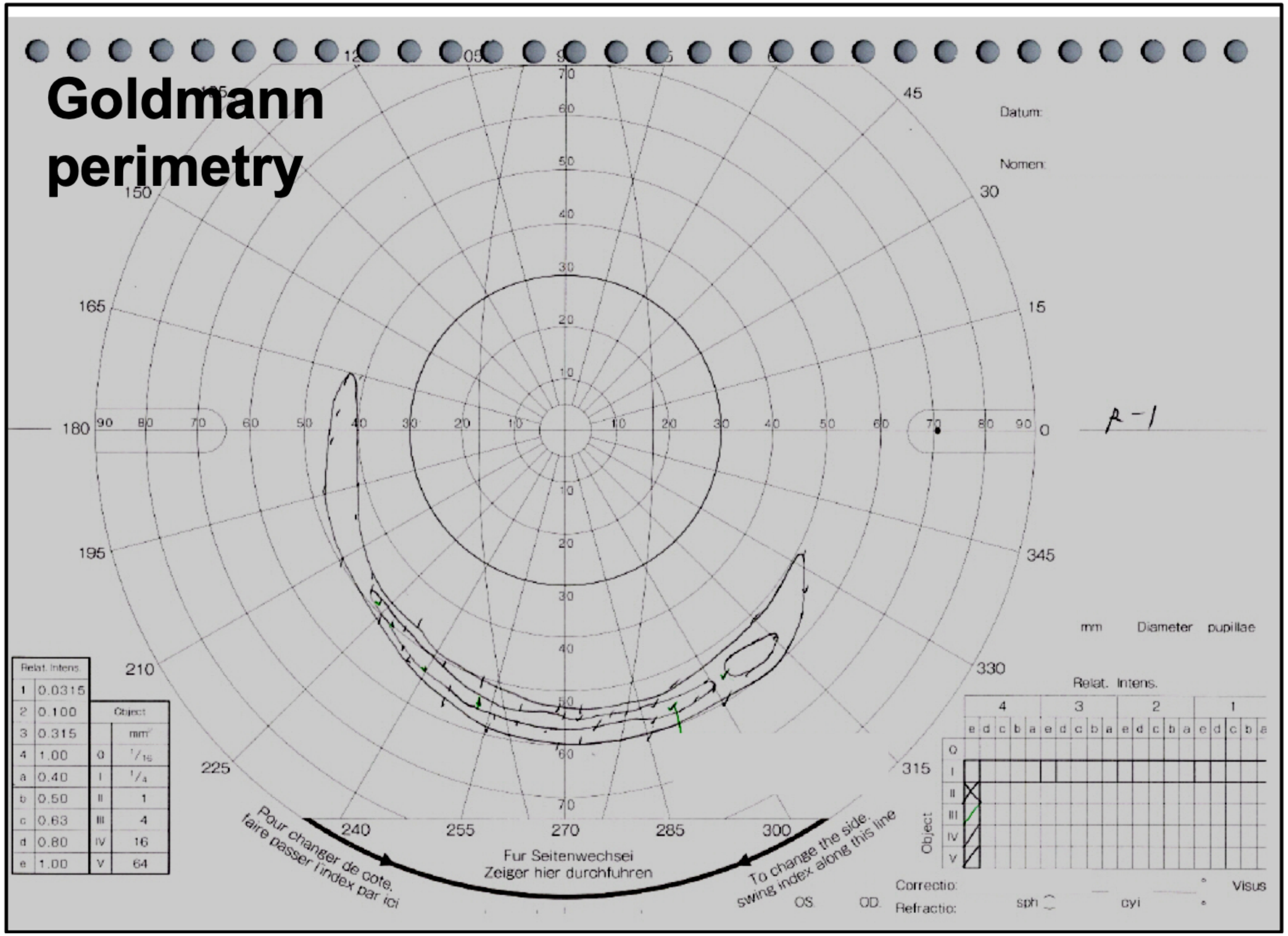

Supplement: Supplementary file 1 — Supplementary Information. [file 41598_2024_53474_MOESM1_ESM.pdf]
